# Macrophages: From Simple Phagocyte to an Integrative Regulatory Cell for Inflammation and Tissue Regeneration—A Review of the Literature

**DOI:** 10.3390/cells12020276

**Published:** 2023-01-11

**Authors:** Andreas Mamilos, Lina Winter, Volker H. Schmitt, Friedrich Barsch, David Grevenstein, Willi Wagner, Maximilian Babel, Karsten Keller, Christine Schmitt, Florian Gürtler, Stephan Schreml, Tanja Niedermair, Markus Rupp, Volker Alt, Christoph Brochhausen

**Affiliations:** 1 Institute of Pathology, University of Regensburg, 93053 Regensburg, Germany; 2 Central Biobank Regensburg, University and University Hospital Regensburg, 93053 Regensburg, Germany; 3 Department of Cardiology, University Medical Centre, Johannes Gutenberg University of Mainz, 55131 Mainz, Germany; 4 German Center for Cardiovascular Research (DZHK), Partner Site Rhine Main, 55131 Mainz, Germany; 5 Medical Center, Faculty of Medicine, Institute for Exercise and Occupational Medicine, University of Freiburg, 79106 Freiburg, Germany; 6 Clinic and Polyclinic for Orthopedics and Trauma Surgery, University Hospital of Cologne, 50937 Cologne, Germany; 7 Department of Diagnostic and Interventional Radiology, University Hospital Heidelberg, 69120 Heidelberg, Germany; 8 Translational Lung Research Centre Heidelberg (TLRC), German Lung Research Centre (DZL), 69120 Heidelberg, Germany; 9 Center for Thrombosis and Hemostasis (CTH), University Medical Center Mainz, Johannes Gutenberg-University Mainz, 55131 Mainz, Germany; 10 Department of Sports Medicine, Medical Clinic VII, University Hospital Heidelberg, 69120 Heidelberg, Germany; 11 Department of Internal Medicine, St. Vincenz and Elisabeth Hospital of Mainz (KKM), 55131 Mainz, Germany; 12 Department of Dermatology, University Medical Centre Regensburg, 93053 Regensburg, Germany; 13 Department for Trauma Surgery, University Hospital Regensburg, 93053 Regensburg, Germany; 14 Institute of Pathology, University Medical Centre Mannheim, Ruprecht-Karls-University Heidelberg, 68167 Mannheim, Germany

**Keywords:** macrophage, plasticity, monocytes, inflammation, tissue regeneration, biomaterials, M1-macrophages, M2-macrophages

## Abstract

The understanding of macrophages and their pathophysiological role has dramatically changed within the last decades. Macrophages represent a very interesting cell type with regard to biomaterial-based tissue engineering and regeneration. In this context, macrophages play a crucial role in the biocompatibility and degradation of implanted biomaterials. Furthermore, a better understanding of the functionality of macrophages opens perspectives for potential guidance and modulation to turn inflammation into regeneration. Such knowledge may help to improve not only the biocompatibility of scaffold materials but also the integration, maturation, and preservation of scaffold-cell constructs or induce regeneration. Nowadays, macrophages are classified into two subpopulations, the classically activated macrophages (M1 macrophages) with pro-inflammatory properties and the alternatively activated macrophages (M2 macrophages) with anti-inflammatory properties. The present narrative review gives an overview of the different functions of macrophages and summarizes the recent state of knowledge regarding different types of macrophages and their functions, with special emphasis on tissue engineering and tissue regeneration.

## 1. Introduction

In the 19th century, the Russian zoologist Ilja (Elie) Metchnikow [1] described a cell, which was able to engulf other cells, bacteria, or solid particles respectively [2,3,4,5]. He called this process phagocytosis. Based on the relatively large diameter of these “eating cells”, the early biologists called them macrophages. After cellular uptake, macrophages kill engulfed cells or organisms. Within the inflammatory process, macrophages, along with neutrophilic granulocytes, are the first line of cellular defense. Metchnikow hypothesized that the inflammatory reaction underwent evolutionary changes like any other biological process. Since macrophages can migrate on their own and integrate foreign bodies, Metchnikow set these cells as the first step in the inflammatory reaction [1]. According to this evolutionary hypothesis, the inflammatory reaction is developed step by step by the involvement of lymphocytes and granulocytes, the cascade of humoral factors of the complement system, and as the latest step, the production of antibodies [6].

Nowadays, our understanding of inflammation and the role of macrophages has dramatically changed. Macrophages are widely accepted to secrete pro-inflammatory or anti-inflammatory cytokines to achieve the orchestration between the different immune cells [7,8,9]. Cytokines are small protein signaling molecules that regulate cells’ growth, differentiation, and function [10]. Today, the term macrophage describes a heterogeneous group of cells with various functions in diverse cellular processes [7]. The first evidence for their heterogeneity was given by Aderem et al., who discovered that macrophages respond to bacterial lipopolysaccharide (LPS) without inducing an inflammatory response via T-cells [11]. Another milestone was the identification of a macrophage subpopulation called “alternatively activated macrophages” (AAM) [12]. Recent studies have shown that macrophages are also involved in synthesizing extracellular matrix (ECM) [13]. Mosser hypothesized that the potency of macrophages to synthesize ECM components gives evidence that these cells potentially have a primary role in tissue repair and not microbial killing [7]. These initial statements give insight into the concept that macrophages play a crucial role in the immune response to pathogens, tissue homeostasis and inflammation, as well as in regeneration and repair [13,14,15]. The present review gives an overview of the different functions of macrophages and summarizes the current state of the literature regarding different types of macrophages and their functions without claiming to be exhaustive.

## 2. The Origin and Formation of Macrophages

**Figure 1:**
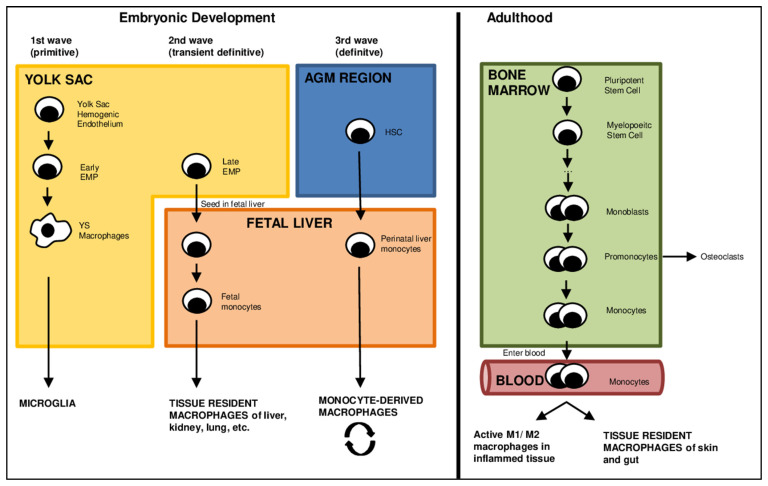
Formation and differentiation of macrophages. Schematic depiction of the differentiation of monocytes during embryonic development unfolding in three sequential waves and adulthood as well as their further differentiation to different subpopulations of macrophages in the tissue [25] (AGM region: aorta-gonad-mesonephros region; EMP: Erythro-Myeloid Progenitors, YS: yolk sac), modified after Corliss et al. [26].

The origin of macrophages has been a matter of debate in recent years. Traditionally, macrophages were seen as descendants of monocytes [16,17]. Monocytes represent a group of white blood cells derived from the myelopoietic stem cells in the bone marrow as all other types of blood and immune cells [18,19,20]. Monocytes are primarily encountered in the blood as circulating cells, but also in the bone marrow and spleen and are incapable of steady state proliferation in these surroundings [21,22]. After their formation in the bone marrow, monocytes enter the blood, where they circulate [10,23] and migrate into various tissues reacting to different stimuli. Such stimuli for monocytic migration may either derive from inflammation or as a result of trauma. After migration into the tissue, monocytes form colonies under the action of chemotactic stimuli (Figure 1) [24]. 

The classical assumption that all macrophages originate from circulating monocytes was discarded years ago. Nowadays, it is well-accepted that hematopoiesis unfolds in three sequential waves, and macrophages undergo self-renewal within the tissue they reside [25]. The comprehensive debate about the origin of macrophages is not focused on in this review and is well demonstrated elsewhere [25,27,28].

## 3. Polarization of Macrophages

Once it became apparent that macrophages not only phagocytize but also express other functions, it was necessary to categorize them further. There is evidence that macrophages are a cell type that can assume various phenotypes based on the stimuli to which they are exposed. Because these cells respond differently to environmental signals [29], the classification centers on their activation mode [14,30,31,32,33]. In this context, macrophages have been categorized into the following subpopulations [31]:

1. Classically activated macrophages (CAM, M1-macrophages)
2. Alternatively activated macrophages (AAM, M2-macrophages)

In 2008, Mosser proposed an alternative classification of macrophages based on three homeostatic activities, which are host defense, wound healing, and immune regulation [22,34]. Furthermore, tumor-associated macrophages have also been identified as a separate group extensively studied in the last few years [35,36,37].

### 3.1. The Plasticity of Macrophages

**Figure 2:**
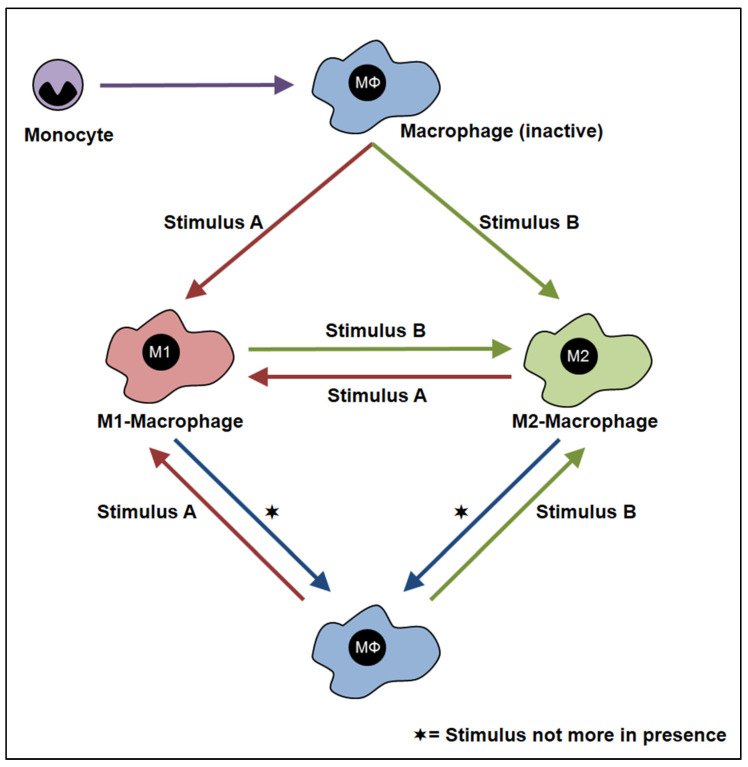
Plasticity of macrophages: a brief schematic depiction of the plasticity of macrophages from an inactive macrophage (MΦ) into either an M1-macrophage or M2-macrophage according to different stimuli. The scheme represents macrophages as an activations state of cells that can be changed along a continuum into the different sub-populations according to various stimuli in the environment. In addition, M1 and M2 macrophages can be turned into inactivated macrophages if there is a lack of stimuli.

The mechanisms resulting in the different macrophage phenotypes are crucial to understanding macrophage subpopulations. This process is called “plasticity” [5] and describes the ability of cells to respond to different microenvironmental influences by displaying diverse functional phenotypes [38,39]. Thus, plasticity results in a polarization of macrophages into different phenotypes assigned to the different subpopulations [14,30,40]. Taking these facts together, it is essential to realize that unlike other cells, which lose their heterogeneity during maturation, macrophages retain their plasticity and transform according to environmental signals [20,29]. Furthermore, there is evidence that the phenotype of polarized M1 and M2 macrophages could be reversed not only experimentally in vitro and in vivo but also in situ (Figure 2) [41,42,43,44]. For instance, in vitro analyses clearly demonstrated the capacity of macrophages to switch between M1 and M2 macrophages using different recombinant cytokines and biologically active substances measured by their CD163 and CD206 expression and their CCL18 and CCL3 production [41]. Furthermore, a switch from M1 to M2 polarized macrophages is described in experimental and human parasite infections [45,46].

In living organisms, the phenomenon of macrophage plasticity ensures that when M1-macrophages have completed the clearance of pathogens and any destroyed surrounding tissue, they transform into M2-macrophages to produce components of the extracellular matrix and simultaneously activate and induce other cells, such as fibroblasts, which also contribute to the formation of extracellular matrix, and thus initiate tissue regeneration [31,47,48]. Another important aspect regarding the plasticity of macrophages concerns their relationship to the modulation of chronic disease and autoimmunity [49]. For a long time, it was taken as a matter of fact that the incomplete or failed switching from one phenotype to another had an impact on chronic inflammation and autoimmune disorders [42]. In chronic venous ulcers, for example, it was shown that macrophages infiltrating the tissue fail to switch from an M1 to an M2 state due to iron overload. Therefore, ROS-mediated DNA damage, fibroblast cellular senescence, and defective tissue repair occur [50]. More recently, it has been demonstrated that even effective macrophage phagocytosis of apoptotic cells is crucially involved in the modulation of chronic inflammatory and autoimmune diseases, which underlines the active regulatory role of macrophages in these pathomechanisms [3,31]. 

Depending on their phenotype, macrophages differ regarding their metabolism being able to switch from an aerobic to an anaerobic state and vice versa. M1-macrophages use glycolysis and pentose phosphate pathways to meet their energy needs. The tricarboxylic acid cycle (TCA) is broken at two points, and itaconate and succinate accumulate. Besides, oxidative phosphorylation and fatty acid oxidation are downregulated. In contrast, M2 macrophages have an intact TCA and increased fatty acid oxidation and oxidative phosphorylation [51]. Another prime example demonstrating the importance of the plasticity of macrophages for their functionality is given by arginine metabolism in the differently polarized macrophages. M1 and M2 macrophages use different arginine-catabolizing enzymes. M1-macrophages metabolize arginine via inducible nitric oxide synthase (iNOS) into nitric oxide (NO) and citrulline, whereas arginase hydrolyzes arginine to ornithine and urea in M2-macrophages. In further downstream pathways, ornithine is broken down into polyamine and proline, which are essential for cellular proliferation and tissue repair [30,52]. M1 and M2 macrophages also differ in their iron metabolism, associated with the respective macrophage function. Iron is essential for bacterial growth as some bacteria obtain energy from the oxidation of divalent iron. Inflammatory M1 macrophages express low levels of hemoglobin receptors (CD163 and CD91), leading to a smaller heme pool within the macrophage. Also, they show high levels of ferritin, an iron storage protein, and low levels of ferroportin, an iron exporting channel affiliated with iron retention resulting in a bacteriostatic effect. In contrast, M2 macrophages express low ferritin levels and high ferroportin levels. The resulting iron release is linked to tissue repair, angiogenesis, and tumor promotion [8,53,54,55]. Therefore, metabolic adaption is a crucial feature of macrophage polarization.

### 3.2. Classically Activated Macrophages (CAM or M1-Macrophages)

CAMs or M1-macrophages are the best-characterized macrophage subpopulation. These cells represent the classical phagocyte [15]. The term “classically activated macrophages” describes those macrophages rising during cell-mediated immune responses [20]. These cells can elicit an effective innate immune response [31]. 

#### 3.2.1. The Activation Process of Classically Activated Macrophages

One of the main activators of M1-macrophages is interferon-gamma (IFN-γ) [56,57]. This cytokine was originally called macrophage-activating factor (MAF). However, today the term MAF is not restricted to IFN-γ but also includes other cytokines and active molecules. IFN-γ has a variety of functions. Thus, it is involved in eliminating viral and intracellular bacterial infections and the mechanisms of tumor control. IFN-γ is an immunomodulator, an immunestimulus, and also can inhibit viral replication directly [58]. In innate immunity, IFN-γ is produced by natural killer T-lymphocytes (NKT) and natural killer (NK) cells. During an antigen-specific immunoreaction, IFN-γ is synthesized by CD8-positive cytotoxic T-lymphocytes (CTL) and CD4-positive TH1-T-helper cells. IFN-γ activates the transcription factors STAT-1 and STAT-2, which bind to gamma-activated sequences (GAS) at various immunological effector genes. As a result, activated macrophages secrete pro-inflammatory cytokines as well as oxygen and nitrogen radicals [7,59,60,61,62,63,64]. Another important activating molecule for macrophages is tumor necrosis factor-alpha (TNF-α), formerly known as cachectin. This molecule is produced by macrophages and is a member of the cytokine family of polypeptide mediators, which also contain interferons and interleukins. TNF-α is an important mediator during inflammation, immune responses, and infectious phenomena. One of the effects of TNF is the initiation of apoptosis, including in tumor cells [65,66,67,68,69]. 

The activation process in classically activated macrophages occurs either in the presence of IFN-γ alone or in combination with other co-stimulating factors [70]. However, a second stimulus is mandatory after the initial stimulation with IFN-γ. In these second stimulating processes, ligands to Toll-like receptors (TLRs) are intimately involved. TLRs bind several microbial components. After ligand-binding, the activated receptor initiates a signal transduction pathway which triggers the production of gene products, which control innate immune responses and further instruct the development of antigen-specific acquired immunity [71,72,73]. The ligands for TLRs are expressed on microorganisms and are known as so-called “pathogen-associated molecular patterns” (PAMPs) [60]. The PAMPs are defined as molecules associated with groups of pathogens that are recognized by cells of the innate immune system. These molecules can be described as low molecular weight signals in a class of microbes. TLRs and other pattern recognition receptors (PRRs) can recognize these PAMP molecules. PAMPs are concerned with the activation of innate immune responses. Essential components of the PAMP molecular family are endotoxins found on the cell membrane of gram-negative bacteria, also known as LPS [74,75,76].

**Figure 3:**
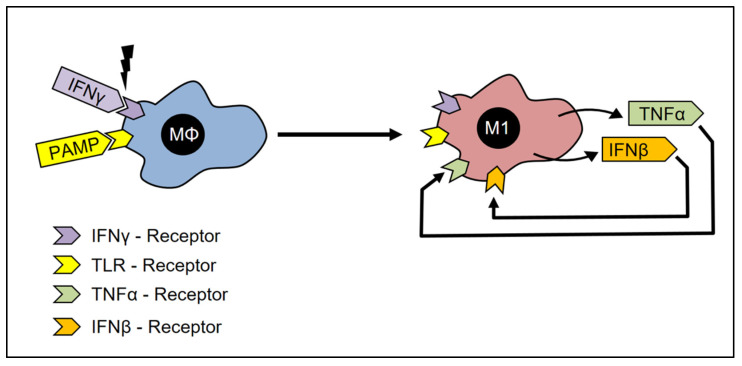
Activation of classically activated macrophages. In the presence of INF-γ-receptor ligands or PAMP, the macrophages adopt the M1 phenotype, which is characterized by the expression of INF-β and TNF-α –receptors. Furthermore, these cells are now capable of synthesizing TNF-α and INF-β and thus achieving self-activation.

The activation of TLRs induces the synthesis of TNF-α, which can act in an autocrine manner to amplify the stimulation of macrophages [77]. Some other TLR-Ligands are able to induce endogenous production of IFN-β [78], which can substitute for IFN-γ [20]. Therefore, after the first activation of CAMs with IFN-γ and LPS, the CAMs are further activated from the endogenously produced TNF-α and IFN-β (Figure 3) [20]. 

M1-macrophages are co-stimulated by additional pro-inflammatory cytokines such as IL-1, IL-12, or other stress signals [48,77]. Stress signals are endogenous factors released from damaged or stressed tissue [79], such as heat-shock proteins, fibronectin fragments, hyaluronan or high-mobility group box 1 proteins [80,81]. Heat-shock proteins, so-called chaperone molecules, are expressed as a reaction to several stressful conditions, such as infections or malignancies [82,83,84]. They are molecules, that detect proteins that had failed to fold or lost their native functional conformation in the cell preventing the aggregation of these proteins [85,86,87]. This phenomenon is described as the stress response [88,89,90]. Fibronectin fragments represent a cleavage product of the extracellular matrix due to the action of metalloproteinases, which are secreted by monocytes during inflammation [91,92]. Fibronectin is a macrophage activator [93]. Hyaluronan is a significant component of the ECM and modulates the inflammatory response [94,95,96]. In this context, larger polysaccharide chains promote anti-inflammatory activity, and smaller to medium size polysaccharide chains have pro-inflammatory properties [90,97,98,99]. High-mobility group box 1 (HMGB-1) is a structural co-factor critical for proper transcriptional regulation in somatic cells and is typically located in the nucleus [100,101]. This molecule, among others, induces inflammation, proliferation, and migration of cells [102,103,104]. HMGB-1 is also passively released by necrotic but not apoptotic cells. Furthermore, it is secreted by activated macrophages [105]. Besides the stimulating stress signals described above, classically activated macrophages can also be co-stimulated by a variety of other molecules [8,16] and hypoxia [106,107,108,109,110].

#### 3.2.2. The Function of Classically Activated Macrophages (CAMs)

The best-described function of M1-macrophages or CAMs is the phagocytosis of pathogens [4,20,111,112,113]. Metchnikoff first described this function more than a hundred years ago [114,115]. Phagocytic activity and the synthesis of toxic agents, such as reactive oxygen and nitrogen species, are reasons why classically activated macrophages belong to the “microbicidal” repertoire of the organism [116,117]. It is important to underline that CAMs express an isoform of iNOS that cannot be detected until the CAMs are activated via IFN-γ and LPS [116,118]. iNOS is an enzyme that synthesizes NO by oxidation of the amino acid L-arginine. NO represents a critical mediator which reacts with superoxide anion (O_2_), resulting in the production of peroxynitrite (ONOO^−^), and these radicals are responsible for the subsequent oxidative damage [119,120,121,122].

To effectively fulfill their function in hostdefense, CAMs secrete various pro-inflammatory cytokines, such as TNFα, IL-1, IL-6, IL-12, and IL-23 [22,123,124,125,126,127]. It is interesting to realize that especially IL-23 [128] but also IL-1 [129,130,131,132], and IL-6 [129,133] have been described as playing an important role in the development of the T-helper cell type 17 (Th17). These cells produce IL-17 [134], which triggers cascades involved in the induction of inflammation and autoimmunity [113,135,136,137,138,139]. In addition to the elimination of pathogens, classically activated macrophages also can present antigens via the MHC-II pathway [7].

**Figure 4:**
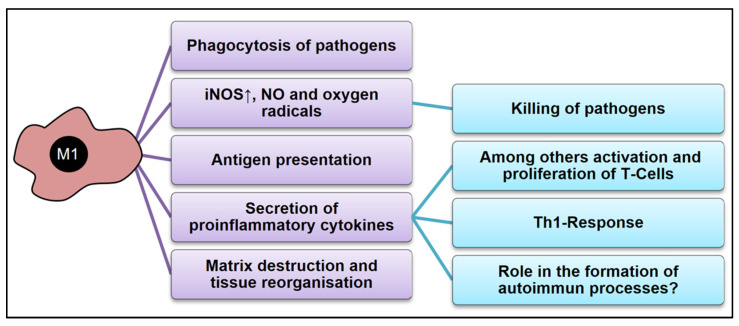
Summary of the basic functions of M1-Macrophages.

As mentioned above, CAMs are also involved in destroying extracellular matrix and tissue reorganization during inflammation or trauma. To achieve this, CAMs produce and secrete various enzymes such as matrix-metalloproteinases (MMPs), macrophage metalloelastase (MMP12), collagenase and hyaluronidase [2,140,141,142,143,144]. MMPs form a group of zinc-dependent proteolytic endoproteinases, which degrade extracellular matrix proteins to support normal tissue remodeling and contribute to tissue destruction during various pathological conditions such as cell-material interactions and tumor cell invasion [145]. Some MMPs also play a role in macrophage polarization. For example, MMP8 has been shown to induce the M2 phenotype via the regulation of TGF-β expression [146,147]. The most important effect of extracellular matrix degradation is the support of macrophage migration through the inflamed tissue to facilitate their functions in clearing cell debris and pathogens (Figure 4).

### 3.3. M2-Macrophages

M2-macrophages are a subpopulation of macrophages that are not activated by the classical pathway via IFN-γ and TNF-α. Gordon et al. introduced the term “alternatively activated macrophages” to characterize a population of macrophages that have to be exposed to IL-4 for activation [60,148]. Also, extracellular nucleotides can influence the differentiation of macrophages into M2-macrophages [149]. M2-macrophages play an important role in various conditions, including immunoregulation, infections, wound healing, and modification of the extracellular matrix by the secretion of proteases and growth factors [150,151]. The group of M2-macrophages includes a minimum of three subpopulations [152,153], which are categorized based on their in vitro activation and polarization pathway:

1. M2a-Macrophages (alternatively activated macrophages, AAM)
2. M2b-macrophages (Type 2—macrophages)
3. M2c-macrophages (deactivated macrophages)

M2-macrophages are capable of releasing the anti-inflammatory cytokine IL-10, thus achieving a Th2-Response [31]. IL-10 is a cytokine with anti-inflammatory activity [154], which has been unequivocally established in various models of infection, inflammation, and even in cancer [155,156,157]. It is a potent inhibitor of antigen presentation and inhibits major histocompatibility complex class II expression and the upregulation of the co-stimulatory molecules CD80 and CD86 [158].

#### 3.3.1. M2a-Macrophages

**Figure 5:**
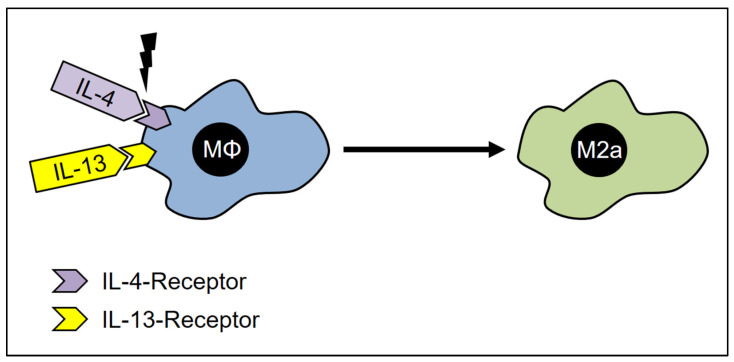
The activation of M2a-Macrophages. In the presence of IL-4- and IL-13- receptor ligands, the macrophages adopt the M2a phenotype, which is characterized by the expression of INF-β and TNF-α –receptors. Furthermore, these cells are now able to synthesize TNF-α and INF-β and thus undergo self-activation.

This subgroup of M2-macrophages is also termed “alternatively activated macrophages” (AAM) [12,159]. These macrophages are characterized by their low expression level of IL-12 [70,159]. In vitro monocytes transform into M2a-macrophages after treatment with IL-4 and IL-13 [14,159,160,161,162,163,164,165]. IL-13 shares a common receptor with IL-4 and exerts similar effects on macrophages [45,166]. IL-4 and IL-13 are cytokines released from various cell sources, including basophils, mast cells, Th2-T-Cells, and innate lymphoid cells. The two interleukins, IL-4 and IL-13, share several structural characteristics and both molecules antagonize the actions of IFN-γ [148,166,167] (Figure 5). After activation by IL-4 and IL-13, M2a-macrophages produce and release IL-1 receptor antagonists, which inhibit IL-1 function [168].

M2a-macrophages are further characterized by their abundant levels of non-opsonic receptors (such as mannose receptor, which is also known as CD206) and the failure (incompetence) to produce NO [45] via the induction of arginase [169], which leads to the generation of ornithine and polyamines [51,70]. They are also characterized by the production of low levels of pro-inflammatory cytokines (IL-1, TNF, and IL-6) and the low expression of the co-stimulatory molecule CD86 [170].

**Figure 6:**
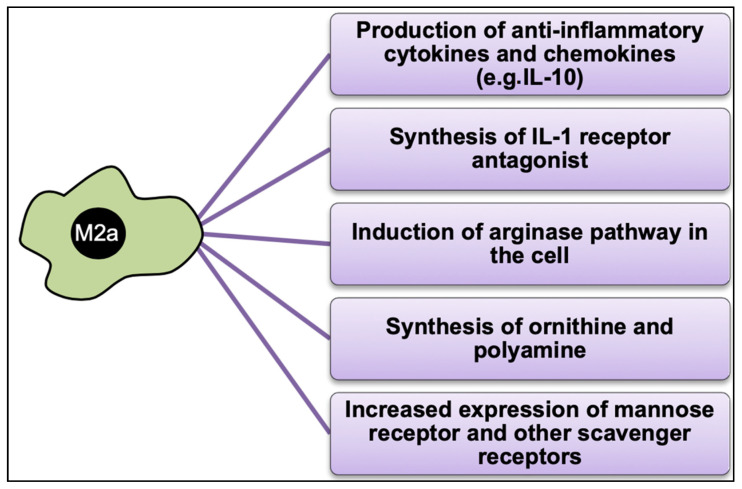
Summary of the function of M2a-macrophages at the cellular level.

Functionally, macrophages are key regulators of fibrosis and resolution. This crucial mechanism is mediated by stabilin-1, a transmembrane glycoprotein expressed by endothelial cells and a subtype of macrophages. It was demonstrated that stabilin-1 expressed by macrophages regulates fibrosis in liver injury [171,172]. In this context, M2a-macrophages have also been described to have a pro-fibrotic potential [167]. In this context, in vitro studies demonstrated that after activation of macrophages with IL-4 or TGF-β, consecutively added myofibroblasts showed an increase in proliferation and the production of fibronectin and collagen I [173,174,175]. However, IL-4-activated macrophages can produce fibronectin and additional matrix proteins, including the TGF-ß-inducible gene H3 (bIG-H3), to a higher degree than classically activated macrophages. Furthermore, the “alternatively activated” macrophages that differentiate in response to IL-4 and IL-13 are involved in Th2-type responses (production of IL-10), including humoral immunity and wound healing [176]. An interesting finding was that activation through IL-4 could lead to an induction of a fusogenic status. This means these macrophages can build multinucleated giant cells (MNGCs) in the presence of other functional components [177,178] (Figure 6). Although some studies show that MNGCs express an M2 rather than M1 phenotype, the exact correlation between macrophage polarization and MNGC formation remains to be further investigated [179,180].

#### 3.3.2. M2b-Macrophages

**Figure 7:**
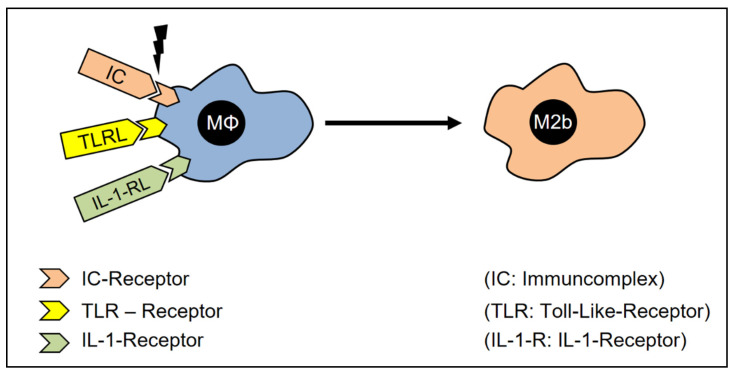
The polarization of M2b-Macrophages. In the presence of immune complexes, TLR-ligands, and IL-1 receptor ligands, the macrophages adopt the M2b phenotype.

To achieve the M2b-macrophage polarization, the macrophages need to be exposed to lipopolysaccharides (agonists of TLR) [181] in the presence of IgG-immune complexes [182,183,184] (Figure 7). Macrophages which, by the time of activation, are exposed to IgG-immune complexes, synthesize large amounts of IL-10 but do not produce IL-12 [182,185].

Despite their high production of inflammatory cytokines and toxic molecules, it could be shown in animal studies that M2b macrophages protect mice against LPS toxicity. Moreover, they promote Th2 differentiation and humoral antibody production [7,70,186,187]. Thus, the M2b-macrophages are more similar to M1 macrophages than alternatively activated macrophages. The M2b are capable of synthesizing NO and have a low arginase activity compared to M2a- und M2c-macrophages. On the other hand, they express the CD86-receptor on their membrane and produce pro-inflammatory cytokines such as TNF, IL-1 and IL-6. One of the basic differences between the M1- and M2b- phenotype is that M2b are able to induce a Th2-response due to the production of IL-10 [20,183], whereas M1-subpopulations induce a Th1-response following their production of IL-12 [188].

#### 3.3.3. M2c-Macrophages

**Figure 8:**
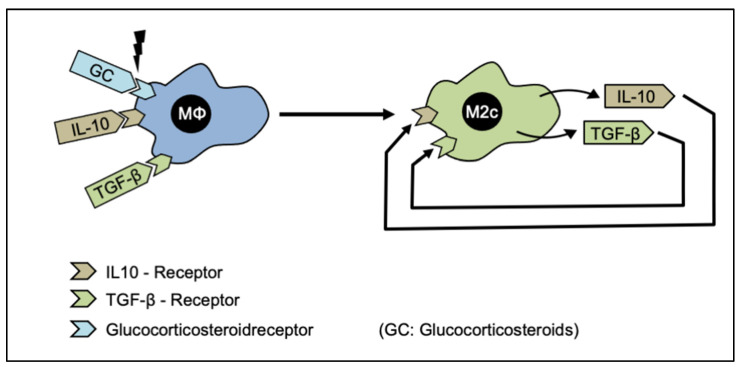
The polarization to M2c-Macrophages. In the presence of glucocorticoids (GC), IL-10 or TGF-β receptor ligands, the macrophages adopt the M2c phenotype, which is capable of synthesizing IL-10 and TGF-β and thus reaching a state of self-activation.

IL-10, TGFβ, or glucocorticoids are required to polarize a macrophage population into the M2c-subgroup [160,162]. After polarization, the M2c-macrophages can produce IL-10 and TGFβ for self-stimulation (autocrine effect). The basic functions of M2c-macrophages are immunosuppression, remodeling of ECM, including matrix deposition, and tissue remodeling [34,189]. Furthermore, the induction of fibrosis triggered by M2c-macrophages has also been reported [31]. Fibrogenesis is a dynamic process in which the synthesis and deposition of ECM components occur as an answer to parenchymal tissue injury. This process plays a pivotal role in multiple physiological and pathological conditions, such as the granulation of wound healing, atherosclerosis, and chronic inflammation [174]. Fibrosis is characterized by the extensive proliferation and activation of tissue fibroblasts, the primary producers of extracellular components [190]. One of the important mediators to modulate proliferation and consecutive ECM components are transforming growth factor β 1 (TGFβ-1) (Figure 8) [191,192]. 

### 3.4. Tumor-Associated Macrophages

In addition to the functions mentioned above, macrophages are essential players in tumorigenesis, tumor promotion, and metastases as they orchestrate cancer-related inflammation and support angiogenesis [193]. During carcinogenesis, circulating monocytes and/ or tissue-resident macrophages are recruited to the tumor niche by mediators secreted by tumor cells and cells of the tumor microenvironment (TME) [193,194,195,196]. These so-called Tumor-Associated Macrophages (TAMs) are a highly plastic, heterogeneous subpopulation of macrophages that cannot be fully captured by the traditional M1/M2 dichotomy [197]. Often, TAMs are referred to as M2d or M2-like macrophages, which might lead to the assumption that only the M2 phenotype occurs in TME. However, M1-like and M2-like macrophages coexist within the TME, secreting opposing factors resulting in their distinct functions [198]. Remarkably, the TAM phenotype is not static and may switch from M1 to M2 as TAMs are sensitive to factors secreted by the TME. One possible explanation for the switch from M1 to M2-like is the expression of adenosine A2A receptors (A2AR) on the surface of M1 macrophages under hypoxic conditions. Adenosine binds to the A2AR suppressing the pro-inflammatory cytokine production (TNF-α, IL-12) and enhancing the secretion of anti-inflammatory and pro-angiogenic factors such as IL-10 and VEGF [187,199]. Furthermore, TAM subsets show a co-expression of M1 and M2 gene signatures, which underlines their broad phenotype spectrum [200].

As described earlier, phenotypical polarization to M1 occurs due to the effect of IFN-y, TNF-α, LPS, and others. The antitumor potential of M1-like TAMs is based on the lysis of tumor cells after phagocytosis or on the secretion of immunostimulatory cytokines and chemokines (e.g., IL-6, IL-12, TNF) which induce inflammation and thus tumor suppression [201]. In contrast, M2-like TAMs are more abundant in the TME and are accepted to be tumor-promoting. As described previously, M2-like TAMs are polarized by IL-4, IL-10, TGFβ-1, and PGE2. Across many cancer entities, the occurrence of M2-like TAMs is linked to numerous tumor-supportive properties such as enhanced tumor cell proliferation, angiogenesis, metastasis, immune suppression, drug resistance, and poor prognosis [202]. The role of TAMs is an extensively reviewed topic in the literature and remains an ongoing field of research. To complete the picture of macrophage heterogeneity, TAMs are mentioned very briefly in the present review but without the claim to be exhaustive. 

## 4. Macrophages and Their Role in Tissue Regeneration

The changing view on macrophages and their different functions in inflammation, wound healing, and regeneration has begun to influence our understanding of their role in different cellular mechanisms in tissue engineering. This concerns not only the biocompatibility of scaffold materials but also the integration, maturation, and preservation of cell-scaffold constructs or induced regeneration. Macrophages are regulators in inflammatory and immunological processes within the tissue, and there are several aspects of tissue engineering in which macrophages play a pivotal role with respect to biomaterials. Relevant examples are the induction of inflammation and host responses as a reaction to biomaterial implants [8,31,48,203,204]. In this context, it is generally accepted that the macrophage is a central element of the inflammatory response, which is practically universally involved in the tissue reaction to implanted biomaterials [205]. 

### 4.1. Immunomodulatory Potential of IL-4

The role of macrophages is regulatory since the phenotypic differentiation to M1 or M2 macrophages is decisive for the secretion of pro- or anti-inflammatory cytokines. As the anti-inflammatory M2 phenotype is associated with improved tissue regeneration, biomaterials should be modified to avoid disadvantageous tissue reactions. Examples of disadvantageous tissue reactions are stenosis in grafts as a result of intima hyperplasia by excessive macrophage infiltration within tissue-engineered vascular grafts [206], but also the formation of peritoneal adhesions or fibrosis after surgical treatment and implantation of biomaterials within the peritoneal cavity [207]. Tan et al. could achieve advantageous tissue reactions in mice by using a bioactive vascular graft coated with IL-4 pushing macrophage polarization toward the M2 phenotype. Consequently, they observed a reduction of foreign body encapsulation and inhibition of neointimal hyperplasia compared to the control group [208]. Recently, resident peritoneal murine macrophages were found to represent an anti-adhesion cell barrier by forming a shield around surgery-induced fibrin clots. Nevertheless, this barrier is frequently inadequate, allowing adhesions to form. By injecting IL-4c, the macrophage barrier was strengthened, and post-operative adhesions were effectively prevented [209]. In the context of macrophage polarization, IL-4 seems to be a promising agent, and further research needs to be performed to establish it in clinical practice.

### 4.2. Importance of Nanomaterial Characteristics 

With a view to the use of various biomaterials as cell carriers, scaffolds, or release systems for signaling molecules and growth factors to trigger tissue regeneration, there is a need to understand the role played by macrophages in the biocompatibility and biodegradation of such implanted materials. If the material is incompatible with the organism, either a severe inflammatory reaction or a foreign body reaction (FBR) is induced, in both of which macrophages are of pathogenetic importance [203]. For instance, Barsch et al. examined whether inflammation and FBR were significantly influenced by the 3D biomaterial design by comparing filamentous fleece and sponge-like biomaterial in a porcine model. Although no statistically significant difference could be found regarding FBR, the sponge-like synthetics showed a significantly lower inflammatory reaction which was quantified based on the density of polymorph-nucleated cells [210]. A further component of biocompatibility is the degradation of biomaterials and the tissue reaction to the degradation products. In this context, macrophages first trigger early acute inflammation, which is mandatory for the elimination of damaged molecules, and then they initiate and regulate the regenerative process [8,211,212,213,214]. However, macrophages are also involved in pathological processes resulting from disturbed wound healing, such as scar formation or delayed and failed regeneration [151]. Taking this pathophysiological function into account, recent strategies are aimed at controlling or modulating macrophages for tissue repair and regeneration. Garash et al. suggest strategies, which include controlled delivery of anti-inflammatory drugs, delivery of macrophages as a component of cellular therapy, controlled release of cytokines that modulate the macrophage phenotype and the design of nanoparticles that exploit the inherent phagocytic character of macrophages [215]. Nanomaterials are an emerging field of interest, but the potential of nanoparticles (NPs) as macrophage regulators has yet to be fully exploited. Ni et al. identified gold NPs as a potential periodontitis treatment option since injection of 45 nm NPs in induced periodontitis in rats resulted in significant anti-inflammatory effects such as M2 polarization [216]. 

### 4.3. Role of Hydrogels and Water-Soluble Substances

Furthermore, Kim and Tabata suggest an enhancement of wound healing by dual release patterns of stromal-derived cell factor-1 and a macrophage recruitment agent from gelatin Hydrogels. The authors demonstrated that culturing macrophages on fibrin gels stimulated the secretion of the anti-inflammatory cytokine interleucin-10 (IL-10) [217]. Hydrogels represent a three-dimensional network filled with water that mimics tissue microenvironment and is therefore considered to be biocompatible material. By conjugating signaling molecules, hydrogels can trigger cells to fulfill distinct functions. For tissue engineering, degradable hydrogels are favored as they can be replaced by growing tissue [218]. Regarding disease and cancer treatment, injectable hydrogels are auspicious biomaterials that can serve as scaffolds and carriers of therapeutic agents [219]. For instance, Xu et al. injected gelatin hydrogel into intracerebral hemorrhage lesions in mice. The researchers demonstrated that inflammation was suppressed in the intervention group. Macrophage polarization was observed to shift towards the M2 phenotype leading to a decline in the secretion of inflammatory cytokines, resulting in reduced neuronal loss and enhanced functional recovery [220]. 

Shiratori et al. showed that drugs could polarize macrophages into different subtypes. For example, Azithromycin, tofacitinib, hydroxychloroquine, and pioglitazone exhibit an anti-inflammatory profile by downregulation of M1 markers and upregulation of some M2 markers [221]. On the other hand, Huang et al. show that synthetic waterborne polyurethane nanoparticles (PU NPs) can inhibit the macrophage polarization toward the M1 phenotype but not toward the M2 phenotype [222]. In contrast, exposure of macrophages to soluble fibrinogen leads to the secretion of large amounts of inflammatory cytokine TNF-α. In conclusion, fibrin exerts a protective effect on macrophages, preventing inflammatory activation. From these findings, the authors concluded that fibrin and fibrinogen might represent key players in regulating macrophage phenotype behavior [217]. A differential regulation of macrophage inflammatory activation by fibrin and fibrinogen was also shown by Hsieh, Smith et al. [223]. 

### 4.4. Role of Iron in Macrophage Polarization

As described above, M1 and M2 macrophages differ in their iron metabolism, so iron oxide nanoparticles (IONPs) are a potent inducer of a switch of polarization. On the one hand, IONPs have been shown to activate macrophages and inhibit tumor growth on their own; on the other hand, IONPs have been used to deliver tumor-suppressing or macrophage-activating biomolecules [224]. Another option to shift TAMs from an M2-like to an M1-like phenotype was reported by Sang et al. They used Sulfur Quantum Dots as a nano trap for free iron ions, which then led to the production of reactive oxygen species and consequently to the reprogramming of macrophages to an M1-like phenotype. The so-activated macrophages could then suppress tumor growth via the activation of immune responses [225].

### 4.5. Influence of the Injury Microenvironment

In each tissue, the injury microenvironment is different. Although the injury triggers, in general, a cascade of more or less the same reactions, the microenvironment and the tissue responses to damage are derived from the tissue composition and the nature of the injury. These unique microenvironments were shown in sterile inflammation against pathogen-mediated inflammation due to the damage-associated molecular patterns recognized by inflammatory cells and not by pathogen-associated molecular patterns. For example, bone regeneration is based on different activities than skeletal muscle regeneration, as reflected by the differences in cytokine, chemokine, and growth factors present during homeostasis and wound healing in these two tissues [226]. If we understand the microenvironment of the inflamed tissue, then it might be easier to develop strategies regarding the reactions between cells and biomaterials. Also, for biomaterial-mediated tissue repair strategies that use endogenous monocyte/macrophage populations, the microenvironment of inflammatory damage can decisively contribute to the criteria for material design. Immunoregenerative materials can be designed to release molecules to enhance or disrupt specific features of the lesion to facilitate repair [227,228], but they should also prioritize the general healing goals of a particular tissue. Firstly, we must understand the properties of a tissue and its responses to injury into consideration of its caused microenvironment and then combine all this knowledge with the polarization of macrophages to achieve proper healing. The cells have to be “guided” in a particular wound environment, according to the damaged tissue [226]. The modulation of macrophages and their phenotype polarization was also shown by Lee et al. They suggest, for example, positive modulation of macrophage phenotype polarization (i.e., towards the regenerative M2 rather than the inflammatory M1 phenotype) with a modified surface, which is essential for the osteogenesis function of Titanium (Ti) bone implants. They showed that nanoscale topographical modification and surface bioactive ion chemistry could positively modulate the macrophage phenotype in a Ti implant surface. They induced the regenerative M2 macrophage phenotype of cells in nanostructured Ti surfaces [229]. Zhu et al. analyzed the modifying role of surface topography on macrophage polarization. The minimal scale of TiO_2_ honeycomb-like structures of 90 nanometers was most effective in stimulating the M2 phenotype. Thus, a favorable anti-inflammatory microenvironment was created, being beneficial for bone formation and osteointegration [230]. Thus, macrophages are also of particular importance for bone and cartilage formation as well as their remodeling [231,232]. From these examples, we can conclude that by controlling the tissue environment and microenvironment, we can control the macrophage behavior and modulate the macrophage phenotype [215].

### 4.6. Role of Macrophages in Angiogenesis

Angiogenesis is a further crucial mechanism that combines macrophages with various processes in tissue engineering. Macrophages take part not only in wound healing as such but also in angiogenesis to support the development and remodeling of vascular networks [110,233,234,235]. Angiogenesis is a multistep process in which macrophages are involved in each step. M2 macrophages secrete proteases (e.g., MMP9) and thus cleave the ECM to create space for the newly forming vessels. In parallel, resting endothelial cells are activated by paracrine stimulation [235]. For this purpose, mainly M1 macrophages secrete pro-angiogenic factors such as VEGF-A, TNF, or FGF2 [236,237,238,239]. After loosening the basement membrane, endothelial sprouting gives rise to new capillaries that migrate toward angiogenic stimuli and then fuse with other sprouts or capillaries to form anastomoses. In “tip cell guidance”, M2 macrophages wrap around the sprouts to facilitate anastomosis formation. Subsequently, the new capillaries maturate, and M2 macrophages remove redundant vessels via phagocytosis [235]. By imitating the physiological process of Angiogenesis, researchers aim to endorse vascularization in tissue engineering scaffolds [240]. In this context, Spiller et al. analyzed the role of macrophage phenotype in the vascularization of scaffolds. They found that M1 and M2c macrophages cause endothelial sprouting and M2a macrophages supported anastomoses. The researchers could control macrophage response by modifying scaffold properties [241]. Another recent study investigated whether reprogramming macrophages with KGM-modified SiO_2_ nanoparticles influences diabetic wound healing. The researchers demonstrated that an M2-like phenotype was linked to angiogenesis, enhanced ECM production, and accelerated wound healing by repressing extensive or persistent inflammation and fibrosis [242]. Targeting angiogenesis in a macrophage-centered treatment approach, therefore, represents a promising target in tissue engineering. 

In conclusion, the differentiation of cells of the monocyte-macrophage lineage into M1 and M2 subpopulations is of major relevance for biomaterial applications in tissue engineering. M1 macrophages are mainly active in the immune system during inflammation. Excessive or prolonged M1 macrophage activation could result in “tissue injury” and thus negatively affect the clinical course of a tissue-engineered implant. On the other hand, M2 macrophages are important for the resolution of inflammation due to their ability to produce anti-inflammatory cytokines. They are also important cells for homeostasis and tissue regeneration. Being able to find the optimal balance between these subpopulations remains a prime challenge in regenerative medicine but holds great promise for the future.

Taking all these findings together, which demonstrate the broad spectrum of macrophage functions, it becomes clear that these cells should be essentially involved in biomaterial- and tissue-engineered-based strategies, and their specific role should be taken into account. Such considerations could open new pathways to modulate the plasticity of macrophages in various tissue engineering approaches.

Metchnikow described macrophages for the first time. They were supposed to phagocytize foreign bodies and bacteria. Now, a hundred years after his death, macrophages are still in trend with a variety of functions and subpopulations. It is our duty to continue to examine them so that we can understand and explain different pathological processes so that we can apply their functions in vitro and in vivo research fields regarding tissue regeneration and engineering, but also in honor and memory of Metchnikow.

## Data Availability

Not applicable.

## List of Abbreviations

A2AR: adenosine A2A receptors; AAM: alternatively activated macrophages; CAM: Classically activated macrophages; CTL: cytotoxic T-lymphocytes; ECM: extracellular matrix; EMP: Erythro-Myeloid Progenitors; FBR: foreign body reaction; GAS: gamma-activated sequences; GC: glucocorticoids; HMGB-1: High-mobility group box 1; HSC: Hematopoietic Stem Cells; HSPs: heat-shock proteins; iNOS: inducible nitric oxide synthase; LIF: leukemia inhibitory factor; LPS: bacterial lipopolysaccharide; MAF: macrophage-activating factor; MMPs: matrix-metalloproteinases; MNGC: multinucleated giant cell; NK: natural killer cells; NKT: natural killer T-lymphocytes; NO: nitric oxide; NP. Nanoparticles; PAMPs: pathogen-associated molecular patterns; PPR: pattern recognition receptors; PU NPs: polyurethane nanoparticles; TAM: tumor-associated macrophages; TCA: tricarboxylic acid cycle; TGFβ-1: transforming growth factor β 1; Th17: T-helper cell type 17; Ti: Titanium; TME: tumor microenvironment; TLRs: Toll-like receptors; TNF-α: tumor necrosis factor-alpha.
